# Association between Undiagnosed Hypertension and Health Factors among Middle-Aged and Elderly Chinese Population

**DOI:** 10.3390/ijerph16071214

**Published:** 2019-04-04

**Authors:** Junmin Zhou, Shu Fang

**Affiliations:** West China School of Public Health, Sichuan University, Chengdu 610041, China; junmin.zhou@scu.edu.cn

**Keywords:** undiagnosed hypertension, health, urban–rural disparity

## Abstract

Undiagnosed hypertension has resulted in significant health and economic burdens. This study sought to investigate the association between health factors and undiagnosed hypertension among hypertensive Chinese and to assess the urban-rural disparity. A total of 6455 diagnosed and undiagnosed hypertensive adults were included. Multivariable logistic regression was conducted to examine the association between health factors and undiagnosed hypertension. The urban–rural disparity was investigated through stratified analysis. Undiagnosed hypertension was prevalent (28.8%), and rural residents were more likely to have undiagnosed hypertension compared to their urban counterparts (30.1% versus 24.7%). Physical examination, healthcare service utilization, body mass index, chronic diseases, headache, and self-rated health status were found to be significantly associated with undiagnosed hypertension. In addition, healthcare service utilization, underweight in body mass index, headache, and self-rating health status were associated with undiagnosed hypertension among the rural sample but not in the urban sample. Undiagnosed hypertension was significantly related to health factors among hypertensive Chinese. The findings provided implications for future hypertension prevention programs. The use of physical examination (e.g., blood pressure measurements) is recommended; special attention may be given to those who are underweight and self-rate their health as good and fair, as they are more likely to be neglected.

## 1. Introduction

Hypertension or high blood pressure remains a daunting global health challenge. The financial cost of hypertension is substantially high across different countries. It is estimated that 31.1% of the global population had hypertension in 2010 [1]. In China, the hypertension prevalence could be as high as 44.7% in 2015–2017 [2]. The increasing hypertension burden is a national public health priority.

However, most people with hypertension are asymptomatic. This leads to a high rate of undiagnosed or undetected hypertension [3]. A previous study indicated that only 44.7% of hypertensive Chinese participants were aware of their diagnosis [2]. The high prevalence of undiagnosed hypertension has resulted in heavy health and economic burdens [2].

To reverse the alarming trend, a large number of studies have been conducted to understand the factors associated with undiagnosed hypertension across different countries [4,5,6,7,8]. Nevertheless, the number of similar studies is minimal in China. Furthermore, hypertension studies in China only include awareness as one part and rarely discuss the associated factors in depth [9,10,11,12,13]. Thus, it appears to be necessary to investigate the relation between undiagnosed hypertension and health factors in the country. Such a step is important, as it may provide significant implications for hypertension prevention.

In addition, a substantial disparity exists between urban and rural China regarding undiagnosed hypertension. Specifically, hypertensive adults living in urban areas are less likely to be undiagnosed as compared to their rural counterparts, even adjusting for socioeconomic status and lifestyle factors [14]. This suggests there are other reasons (e.g., health factors) for the disparity.

Therefore, the primary objective of the study was to investigate the association between health factors and undiagnosed hypertension among hypertensive Chinese, and the secondary objective was to assess the urban–rural disparity.

## 2. Methods

### 2.1. Study Population and Design

The data used came from the Chinese Health and Retirement Longitudinal Study (CHARLS) conducted in 2015. The participants were Chinese community residents over the age of 45. The aim of the CHARLS is to address the needs of scientific research related to the rapid ageing of China’s population and to support scientific research about middle-aged and elderly Chinese people. Detailed information about the CHARLS design, method, and recruitment strategy has been described elsewhere [15].

**Figure 1:**
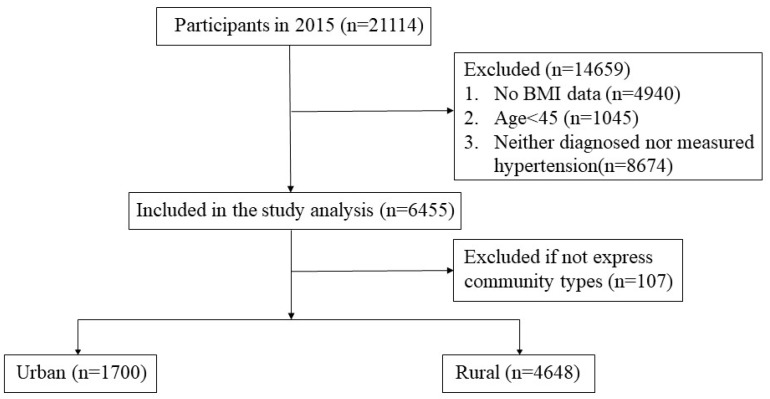
Sample included in the analysis.

As shown in Figure 1, a total of 21,114 individuals were interviewed in 2015, and 6455 of them were included in the analysis. Participants were excluded if they (1) lacked physical examination data; (2) were younger than 45 years old; (3) were neither diagnosed nor measured as hypertensive.

The data are the secondary data from CHARLS and are accessible to be downloaded publicly at http://charls.pku.edu.cn/zh-CN/page/data/2015-charls-wave4. All participants provided informed consent before receiving the investigation. The ethical approval of data collection was from the Biomedical Ethics Review Committee of Peking University (IRB00001052-11015) [16].

### 2.2. Measurements

#### 2.2.1. Measures of Hypertension Prevalence

##### Diagnosed Hypertension

Participants were asked, “Have you been diagnosed with hypertension by a doctor?” Participants who said “yes” to the question were defined as having diagnosed hypertension. Participants who responded “no” were regarded as not having diagnosed hypertension.

##### Measured Hypertension

Participants received blood pressure (BP) measurement three times in the physical examinations. The Omron HEM-7200 monitor was used. A participant was considered as having measured hypertension if his or her mean systolic blood pressure (SBP) was ≥140 mmHg and/or mean diastolic blood pressure (DBP) was ≥90 mmHg [17].

##### Total

Participants who had diagnosed hypertension and/or had measured hypertension were defined as having hypertension.

##### Undiagnosed Hypertension

Participants who were hypertensive but did not report having been told by a doctor that they have hypertension were classified as having undiagnosed hypertension.

### 2.3. Health Variables

Physical examination was classified as “Yes” (physically examined in last two years) or “No”. Healthcare service utilization was classified as “Yes” (visited a public hospital, private hospital, public health center, clinic, or health worker’s or doctor’s practice, or were visited by a health worker or doctor for outpatient care in the last month) or “No”.

Body mass index (BMI) was calculated. Underweight was defined as BMI <18.50, normal weight was 18.50–23.99, overweight was 24.00–27.99, and obesity was 28.00 and over [18]. Participants who had been diagnosed with diabetes, dyslipidemia, chronic lung diseases, or heart disease were defined as having chronic diseases (other than hypertension).

### 2.4. Covariates

Based on national regulations, community types were categorized as “Urban” (main city zone/combination zone between urban and rural areas/the town center/ZhenXiang area/special area) and “Rural” (township central/village) [19]. Smoking and alcohol consumption were categorized as “Current/Former” and “Never”. Physical activity was measured as a dichotomous variable indicating whether respondents participated in walking or any activities requiring hard/high or moderate intensity physical effort for at least 10 minutes continuously during a usual week. Social activity was measured by asking participants if they participated in any social activities such as interacting with friends, going to community club, doing voluntary or charity work, etc., in the last month. Annual household income was categorized into tertiles: 0 to ≤4000, 4001 to 25,000, and >25,000 CNY (Chinese Yuan Renminbi—RMB: ¥; Yuan—US Dollar exchange rate was approximately 6.2 Yuan per US dollar in 2015). It was calculated by aggregating income across the respondent and his/her family members. Single imputation techniques were used to derive all components of household income.

### 2.5. Statistical Analysis

Mean and numbers (proportions) were used to describe continuous variables and categorical variables, respectively. Differences on characteristics of continuous variables and categorical variables between the rural sample and the urban sample were examined by *t* tests and χ^2^ tests. Multivariable logistic regressions were conducted to examine the relationship between health-related variates and undiagnosed hypertension. In addition to the crude models, multivariable adjusted logistic regression analyses were used, adjusting for potential confounders including age, gender, community types, marital status, educational attainment, annual household income, employment, smoking, alcohol consumption, physical activity, and social activity. To further explore community type differences in the association between health-related variates and undiagnosed hypertension, stratified analysis by community types was carried out. The odds ratios (OR) and the corresponding 95% confidence intervals (95% CI) were calculated. The association was considered to be statistically significant if the two-sided *p* value was <0.05. SPSS version 21 (IBM, Armonk, NY, USA) was used to perform all statistical analyses [20].

## 3. Results

Table 1 indicates descriptive statistics for all variables among the whole sample, the urban sample, and the rural sample. The urban sample and the rural sample had statistically significant differences (*p* < 0.001) in undiagnosed hypertension, educational attainment, annual household income, employment, social activity, BMI, chronic disease (other than hypertension), headache, self-rated health status, and physical examination.

Overall, undiagnosed hypertension was prevalent in the sample (28.8%), and rural residents were more likely to have undiagnosed hypertension (30.1%) compared to their urban counterparts (24.7%). The mean age in the sample was 63.1. Half of the sample was men, and the other half was women. A majority of the sample (79.3%) were married. The urban–rural disparity in education attainment was appreciable. Within the urban sample, 48.7% of participants received middle school or above education, while the corresponding percentage was only 22.4% among rural participants. Similarly, urban participants were more likely to report higher annual household income than rural participants. For instance, half of the urban sample reported an income of over 25,000 CNY, while only 24.7% of the rural sample was in this category. Most of the urban sample was not employed (62.7%), but most of rural sample was employed (67.1%). Urban residents were more likely to participate in social activities compared to their rural counterparts (62.8% versus 51.4%). Urban residents had a higher BMI compared to their rural counterparts, as they were less likely to be underweight (2.4% versus 4.7%) and normal weight (31.8% versus 42.0%) but more likely to be overweight (42.9% versus 36.2%) and obese (22.9% versus 17.2%). Concerning chronic diseases other than hypertension, urban residents were more likely to report (54.4% versus 41.8%). Rural residents were more likely to have headache (18.2% versus 12.3%) and poor self-rated health status (23.7% versus 16.4%) than their urban counterparts. In the urban sample, 59.0% of participants had a physical examination in the last two years, but this number was only 44.9% in the rural sample.

Table A1 in the Appendix A shows the specific characteristics of diagnosed hypertensives and undiagnosed hypertensives in the sample, urban, and rural residents.

Table 2 presents the multivariable logistic regressions assessing the associations between health-related variables and undiagnosed hypertension before and after adjusting for potential confounding variables. In the adjusted models, those who had a physical examination in the last two years were less likely to be undiagnosed compared to those who did not (OR = 0.64, 95% CI = 0.56–0.72), and this association was also significant among both urban and rural samples (OR = 0.69, 95% CI = 0.53–0.90; OR = 0.62, 95% CI = 0.53–0.72 respectively). With regard to healthcare service utilization, those who used a healthcare service in the last month were less likely to be undiagnosed (OR = 0.73, 95% CI = 0.62–0.86), but this association was only significant in the rural sample (OR = 0.69, 95% CI = 0.57–0.83), not in the urban sample. Compared to those had normal weight, underweight participants had a higher probability of undiagnosed hypertension (OR = 1.55, 95% CI = 1.15–2.08). Again, this association was found significant only among rural residents (OR = 1.50, 95% CI = 1.09–2.07), not in urban people. Nevertheless, being overweight (OR = 0.69, 95% CI = 0.60–0.79) or obese (OR = 0.45, 95% CI = 0.37–0.55) was less likely to be undiagnosed compared to those who had normal weight. No urban–rural disparity was found in this association. Reporting chronic diseases other than hypertension was associated with a lower probability of undiagnosed hypertension in the whole sample (OR = 0.44, 95% CI = 0.38–0.50), the urban sample (OR = 0.44, 95% CI = 0.33–0.57), and the rural sample (OR = 0.44, 95% CI = 0.38–0.52). Those who reported a symptom of headache were less likely to have undiagnosed hypertension (OR = 0.61, 95% CI = 0.50–0.75). This association was only significant among the rural sample (OR = 0.56, 95% CI = 0.45–0.70), not in the urban sample. Notably, participants with better self-rated health status had a greater tendency to have undiagnosed hypertension. Specifically, the odds of having undiagnosed hypertension for those with fair self-rated health status were 1.55 (95% CI = 1.29–1.87) times higher than those with poor health status within the whole sample and 1.57 (95% CI = 1.28–1.92) times in rural residents; when referring to good self-rated health status versus poor health status, the probability of having undiagnosed hypertension was larger (OR = 2.43, 95% CI = 1.96–3.00 in the whole sample, OR = 3.09, 95% CI = 1.88–5.06 in the urban sample, and OR = 2.26, 95% CI = 1.79–2.87 in the rural sample).

## 4. Discussion

In the study, we assessed the prevalence of undiagnosed hypertension among the population with hypertension and the relationships between undiagnosed hypertension and health-related variables. Physical examination, healthcare service utilization, BMI, chronic diseases, headache, and self-rated health status were all found to be significantly associated with undiagnosed hypertension. In addition, certain urban–rural disparities were detected, as healthcare service utilization, underweight in BMI, headache, and self-rating health status as fair were associated with undiagnosed hypertension among the rural sample but not in the urban sample.

In the study, 28.8% of the hypertensive participants had undiagnosed hypertension, and it was more prevalent in rural participants than in their urban counterparts. This was significantly lower than the findings reported by most existing literature, which found that “rule of halves” applies to a number of populations (including Chinese and non-Chinese) [12,21]. The “rule of halves” means approximately half of hypertensive participants are unaware of their hypertension status. Nevertheless, a recent study reported that the unawareness of hypertension among hypertensive Chinese in urban areas was 25.5% and proposed that enhancement in the education level and preventive measures from the government and healthcare professionals might improve the situation [9]. However, the disparity between urban and rural China in our study still exists, and this warrants further attention.

Physical examination was negatively associated with undiagnosed hypertension. This was partially in line with previous research, which suggested that frequency of blood pressure measurements is related to hypertension awareness [13]. As an important part of physical examination, BP measurement is essential in the diagnosis of hypertension. Therefore, people are suggested to take active part in regular health checks or physical examinations (including BP measurement).

Those who reported healthcare service utilization in the last month were less likely to have their hypertension undiagnosed. This was consistent with a previous study that found that the utilization of health care is negatively associated with the percentage of unawareness of hypertension among adults [22]. The findings were not surprising since increased contact with healthcare providers leads to increased opportunities of detecting hypertension. However, the association was found to be significant only among the rural sample, not in the urban sample. This could be because urban residents have better health literacy than their rural counterparts [23], thus they may not need to interact with healthcare providers to understand their blood pressure. Therefore, the increased healthcare service utilization in the urban sample did not necessarily serve as a protective factor any longer.

Being overweight and obese were associated with lower probability of undiagnosed hypertension, which was in agreement with other studies [9,10]. Overweight and obesity positively impact the frequency of BP measurements and thus increase the hypertension awareness [24]. Nevertheless, our results found that the underweight were more likely to be undiagnosed compared to those who had normal weight in both the whole sample and the rural sample. Underweight populations were traditionally considered to be groups with low risk of hypertension, thus more likely to be neglected and undiagnosed [25]. The correlation was significant only in the rural sample, possibly because urban participants have better access to health care than their rural counterparts, thus urban respondents who are underweight have more opportunities to be diagnosed [11].

Having chronic diseases (other than hypertension) was negatively correlated with undiagnosed hypertension. This was partially in accordance with existing studies, which found that diabetes and dyslipidemia are associated with hypertensive awareness among all participants with hypertension [13]. People with other chronic conditions have more opportunities to interact with healthcare providers or health information, thus are more likely to be aware of their hypertensive status.

Participants who complained of headaches were less likely to be undiagnosed. Headache is usually a leading symptom of hypertension and is potentially caused by severe hypertension [26]. The significant association in the rural sample suggested that rural hypertensive participants might not get diagnosed until they seek assistance with their headaches, possibly due to worse health literacy or worse access to healthcare [11,23].

Contrary to the traditional view that self-rated health is a valid health status indicator [27], those who rated their health as good and fair were more likely to be undiagnosed compared to participants who self-rated poor health. The finding was consistent with previous ones [28,29], and raised the question about the validity of self-rated health as a proxy measure to objective health outcomes—at least for hypertension. This traditional view may make the groups vulnerable to being neglected in hypertension prevention programs. Future studies are needed to corroborate the finding.

The study had several limitations. First, inferring causality was not possible in the cross-sectional design. Second, self-reported data (e.g., diagnosed hypertension) might have incurred recall bias. Third, family history of hypertension and dietary factors were not included due to the unavailability of the data. Forth, the blood pressure was measured three times in physical examinations in the study, which was different from the 2017 Hypertension Clinical Practice Guidelines recommending an average of ≥2 readings obtained on ≥2 occasions [30]. Fifth, health service utilization was measured in one month, which could have been too short. Despite these limitations, our study was unique, as it comprehensively examined the health factors associated with undiagnosed hypertension and provided cues for future hypertension prevention programs.

### Applications

The use of a physical examination (e.g., BP measurements) at least once every two years is recommended; hypertension control programs should be prioritized; special and more attention may be given to those who are underweight and self-rate their health as good and fair, as they are more likely to be neglected.

## 5. Conclusions

The study established the association between undiagnosed hypertension and health factors among hypertensive Chinese. The findings provided implications for future hypertension prevention programs by suggesting focusing on certain groups.

## Figures and Tables

**Table 1:** Characteristics of participants in the sample (*N* = 6455), urban (*n* = 1700), and rural (*n* = 4648) residents in China, 2015.

Variable	Whole Sample (6455)	Urban Sample (1700)	Rural Sample (4648)	*p* Value
	Number (Percentage) or Mean ± SD	Number (Percentage) or Mean ± SD	Number (Percentage) or Mean ± SD	
Variable of Interest				
Hypertension				<0.001
Diagnosed	4530 (71.2)	1276 (75.3)	3246 (69.9)	
Undiagnosed	1828 (28.8)	418 (24.7)	1400 (30.1)	
Individual characteristics				
Age	63.10 ± 9.83	63.07 ± 9.85	63.16 ± 9.84	0.76
Gender				0.25
Men	3105 (48.1)	839 (49.4)	2220 (47.8)	
Women	3350 (51.9)	858 (50.6)	2428 (52.2)	
Marital status				0.89
Married	5119 (79.3)	1043 (62.8)	3682 (79.2)	
Unmarried	1336 (20.7)	617 (37.2)	966 (20.8)	
Socioeconomic gradient				
Educational attainment				<0.001
Did not finish primary school	2856 (47.0)	433 (28.0)	2378 (53.8)	
Elementary school	1441 (23.7)	360 (23.3)	1053 (23.8)	
Middle school or above	1777 (29.3)	754 (48.7)	989 (22.4)	
Annual household income				<0.001
≤4000	2246 (34.8)	304 (17.9)	1899 (40.9)	
4001–25,000	2177 (33.7)	545 (32.1)	1601 (34.4)	
>25,000	2032 (31.5)	848 (50.0)	1148 (24.7)	
Employment				<0.001
Yes	3813 (59.2)	632 (37.3)	3111 (67.1)	
No	2630 (40.8)	1063 (62.7)	1528 (32.9)	
Health behaviors				
Smoking status				0.17
Never	3575 (55.5)	962 (56.8)	2543 (54.8)	
Current/Former	2865 (44.5)	732 (43.2)	2094 (45.2)	
Alcohol consumption				0.10
Never	4221 (65.5)	1080 (63.7)	3063 (66.0)	
Current/Former	2224 (34.5)	615 (36.3)	1577 (34.0)	
Physical activity				0.36
No	3765 (58.3)	1007 (59.3)	2696 (58.0)	
Yes	2690 (41.7)	690 (40.7)	1952 (42.0)	
Social activity				<0.001
Yes	3424 (54.4)	1043 (62.8)	2326 (51.4)	
No	2867 (45.6)	617 (37.2)	2198 (48.6)	
Health-related variables				
BMI (body mass index)				<0.001
Under weight	263 (4.1)	40 (2.4)	217 (4.7)	
Normal weight	2541 (39.4)	540 (31.8)	1951 (42.0)	
Overweight	2443 (37.8)	728 (42.9)	1681 (36.2)	
Obese	1208 (18.7)	389 (22.9)	799 (17.2)	
Chronic disease (other than hypertension)				<0.001
No	3533 (54.9)	770 (45.6)	2700 (58.2)	
Yes	2904 (45.1)	918 (54.4)	1939 (41.8)	
Headache				<0.001
No	5374 (83.3)	1488 (87.7)	3801 (81.8)	
Yes	1081 (16.7)	209 (12.3)	847 (18.2)	
Self-rated health status				<0.001
Fair	3531 (56.2)	969 (58.4)	2503 (55.4)	
Good	1383 (22.0)	419 (25.2)	942 (20.9)	
Poor	1367 (21.8)	272 (16.4)	1070 (23.7)	
Physical examination				<0.001
Yes	3129 (48.6)	997 (59.0)	2080 (44.9)	
No	3306 (51.4)	693 (41.0)	2555 (55.1)	
Healthcare service utilization				0.19
Yes	1372 (21.3)	339 (20.0)	1003 (21.6)	
No	5067 (78.7)	1353 (80.0)	3635 (78.4)	
Measured BP (blood pressure)				
SBP (systolic BP)	143.33 ± 19.96	143.13 ± 19.14	143.84 ± 20.10	0.20
DBP (diastolic BP)	82.46 ± 12.16	81.95 ± 12.03	82.83 ± 12.19	0.01

**Table 2:** The odds ratios (OR) of health-related variables on undiagnosed hypertension for multivariable logistic regressions among sample (*N* = 6455), urban (*n* = 1700), and rural (*n* = 4648) residents in China, 2015.

Variable	Whole Sample		Urban Sample		Rural Sample	
	Crude Model	Adjusted Model	Crude Model	Adjusted Model	Crude Model	Adjusted Model
Physical examination						
No	Ref	Ref	Ref	Ref	Ref	Ref
Yes	0.54 (0.48–0.60) ***	0.64 (0.56–0.72) ***	0.58 (0.47–0.72) ***	0.69 (0.53–0.90) **	0.54 (0.47–0.61) ***	0.62 (0.53–0.72) ***
Healthcare service utilization						
No	Ref	Ref	Ref	Ref	Ref	Ref
Yes	0.53 (0.46–0.61) ***	0.73 (0.62–0.86) ***	0.59 (0.44–0.81) **	1.10 (0.77–1.56)	0.50 (0.43–0.60) ***	0.69 (0.57–0.83) ***
BMI						
Normal weight	Ref	Ref	Ref	Ref	Ref	Ref
Under weight	1.26 (0.97–1.63)	1.55 (1.15–2.08) **	1.44 (0.75–2.78)	1.78 (0.80–3.97)	1.21 (0.91–1.61)	1.50 (1.09–2.07) *
Overweight	0.66 (0.58–0.75) ***	0.69 (0.60–0.79) ***	0.69 (0.54–0.89) **	0.70 (0.53–0.93) *	0.66 (0.57–0.76) ***	0.68 (0.58–0.80) ***
Obese	0.39 (0.33–0.47) ***	0.45 (0.37–0.55) ***	0.36 (0.26–0.51) ***	0.41 (0.28–0.62) ***	0.42 (0.35–0.51) ***	0.46 (0.37–0.58) ***
Chronic disease (other than hypertension)						
No	Ref	Ref	Ref	Ref	Ref	Ref
Yes	0.30 (0.26–0.34) ***	0.44 (0.38–0.50) ***	0.29 (0.23–0.37) ***	0.44 (0.33–0.57) ***	0.30 (0.26–0.35) ***	0.44 (0.38–0.52) ***
Headache						
No	Ref	Ref	Ref	Ref	Ref	Ref
Yes	0.43 (0.36–0.51) ***	0.61 (0.50–0.75) ***	0.51 (0.35–0.76) **	0.91 (0.57–1.44)	0.39 (0.32–0.47) ***	0.56 (0.45–0.70) ***
Self-rated health status						
Poor	Ref	Ref	Ref	Ref	Ref	Ref
Fair	2.03 (1.72–2.39) ***	1.55 (1.29–1.87) ***	1.85 (1.26–2.73) **	1.55 (0.99–2.45)	2.17 (1.81–2.59) ***	1.57 (1.28–1.92) ***
Good	3.83 (3.19–4.58) ***	2.43 (1.96–3.00) ***	4.55 (3.03–6.82) ***	3.09 (1.88–5.06) ***	3.77 (3.07–4.62) ***	2.26 (1.79–2.87) ***

* *p* < 0.05, ** *p <* 0.01, *** *p* < 0.001.

## Appendix A

**Table A1:** Characteristics of diagnosed hypertensives and undiagnosed hypertensives in the sample (*N* = 6455), urban (*n* = 1700), and rural (*n* = 4648) residents in China, 2015.

Variable	Whole Sample (6455)		Urban Sample (1700)		Rural Sample (4648)	
	Percentage or Mean ± SD		Percentage or Mean ± SD		Percentage or Mean ± SD	
	Diagnosed Hypertensives	Undiagnosed Hypertensives	Diagnosed Hypertensives	Undiagnosed Hypertensives	Diagnosed Hypertensives	Undiagnosed Hypertensives
*N*	4530	1828	1275	419	3246	1400
Variable of Interest						
Individual characteristics						
Age	63.25 ± 9.54	62.89 ± 10.56	63.30 ± 9.68	62.43 ± 10.31	63.21 ± 9.48	63.03 ± 10.64
Gender						
Men	45.4	54.9	45.7	59.9	45.3	53.5
Women	54.6	45.1	54.3	40.1	54.7	46.5
Marital status						
Married	79.6	78.1	80.4	76.6	79.4	78.9
Unmarried	20.4	21.9	19.6	23.4	20.6	21.1
Socioeconomic gradient						
Educational attainment						
Did not finish primary school	47.7	45.8	29.0	25.7	54.8	51.5
Elementary school	23.0	25.3	22.9	24.3	23.0	25.7
Middle school or above	29.3	28.9	48.1	50.0	22.2	22.8
Annual household income						
≤4000	34.8	34.6	18.4	16.9	41.2	39.9
4001–25,000	33.5	34.6	32.1	32.0	34.0	35.4
>25,000	31.7	30.7	49.6	51.1	24.7	24.6
Employment						
Yes	56.5	65.4	34.1	46.8	65.3	71.1
No	43.5	34.6	65.9	53.2	34.7	28.9
Health behaviors						
Smoking status						
Never	57.7	49.8	59.5	48.4	56.8	50.2
Current/Former	42.3	50.2	40.5	51.6	43.2	49.8
Alcohol consumption						
Never	68.5	57.8	67.0	54.2	69.0	59.0
Current/Former	31.5	42.2	33.0	45.8	31.0	41.0
Physical activity						
No	58.6	57.8	59.8	57.5	58.1	57.7
Yes	41.4	42.2	40.2	42.5	41.9	42.3
Social activity						
Yes	44.4	48.2	37.1	37.5	52.7	48.5
No	55.6	51.8	62.9	62.5	47.3	51.5
Health-related variables						
BMI						
Under weight	3.4	5.7	1.9	3.8	4.0	6.3
Normal weight	35.8	48.1	28.9	40.8	38.4	50.3
Overweight	39.2	34.7	43.1	42.0	37.8	32.5
Obese	21.6	11.4	26.0	13.4	19.8	10.9
Chronic disease (other than hypertension)						
No	46.8	74.8	38.2	67.9	50.2	76.8
Yes	53.2	25.2	61.8	32.1	49.8	23.2
Headache						
No	80.4	90.6	86.1	92.4	78.1	90.2
Yes	19.6	9.4	13.9	7.6	21.9	9.8
Self-rated health status						
Fair	56.6	55.1	60.9	50.6	55.0	56.5
Good	17.8	32.6	20.0	40.9	16.9	30.2
Poor	25.6	12.3	19.0	8.5	28.1	13.3
Physical examination						
Yes	53.1	37.8	62.4	48.9	49.4	34.4
No	46.9	62.2	37.6	51.1	50.6	65.6
Healthcare service utilization						
Yes	24.0	14.3	22.0	14.4	24.8	14.3
No	76.0	85.7	78.0	85.6	75.2	85.7
Measured BP						
SBP	141.22 ± 20.04	149.44 ± 18.26	140.65 ± 18.66	150.27 ± 18.81	141.11 ± 20.53	149.26 ± 17.96
DBP	81.07 ± 12.12	86.25 ± 11.43	80.27 ± 11.89	86.76 ± 11.10	81.39 ± 12.19	86.09 ± 11.53
